# Association between serum lipid profile, body mass index and osteoporosis in postmenopausal Sudanese women

**DOI:** 10.4314/ahs.v22i3.43

**Published:** 2022-09

**Authors:** Asgad Osman Alfahal, Abdalla Eltoum Ali, Gadallah Osman Modawe, Wael Mohialddin Doush

**Affiliations:** 1 AlzaiemAlazhari University, College of Medical Laboratory Sciences, Department of Clinical Chemistry, Khartoum, Sudan; 2 Omdurman Islamic University, Faculty of Medicine and Health Sciences, Department of Biochemistry, Khartoum, Sudan; 3 Omdurman Islamic University, Faculty of Medicine and Health Sciences, Department of Surgery, Khartoum, Sudan

**Keywords:** Osteoporosis, Postmenopausal women, Serum lipid profile, Sudan

## Abstract

**Background:**

Epidemiological observations suggest links between osteoporosis and the risk of acute cardiovascular events. Whether the two clinical conditions are linked by common pathogenic factors or atherosclerosis per se remains incompletely understood. The reduction of bone density and osteoporosis in postmenopausal women contributes to elevated lipid parameters and body mass index (BMI).

**Objective:**

To investigate the relationship between serum lipid profile, BMI and osteoporosis in postmenopausal women.

**Materials and Methods:**

A prospective analytical case control-study conducted in Khartoum north hospital at Khartoum city, capital of the Sudan from April 2017 to March 2018 after ethical approval obtained from the local Research Ethics Committee of Faculty of Medical Laboratories, Alzaeim Alazhary University on the committee meeting number (109) on Wednesday 15th February 2017. A written informed consent was obtained from all participants to participate in the study.

Two hundred postmenopausal women were enrolled in the study. The age was studied in one hundred osteoporosis postmenopausal women as a case group and one hundred non-osteoporosis postmenopausal women as control group. The serum lipid profiles were estimated using spectrophotometers (Mandry) and BMI calculated using Quetelet index formula. The data were analysed using SPSS version 16.

**Results:**

The BMI, serum total cholesterol, triglyceride, HDL and LDL in case group respectively were (24.846±2.1647, 251.190±27.0135 mg/dl, 168.790 ±45.774 mg/dl, 50.620 ± 7.174 mg/dl, 166.868 ±28.978 mg/dl). While the BMI, serum total cholesterol, triglyceride, HDL and LDL in control group respectively were (25.378 ±3.8115, 187.990 ± 26.611 mg/dl, 139.360±20.290 mg/dl, 49.480 ±4.659 mg/dl, 111.667 ±28.0045 mg/dl). All serum lipid profiles significantly increased (p=0.000) in the case group compared to the control group, except serum HDL was insignificant different between the case and control group and also BMI was insignificant different between the case and control group. There was a positive Pearson's correlation between BMD and serum total cholesterol (r= 0.832, P<0.01), serum LDL (r = 0.782, P<0.01) and serum triglyceride (r = 0.72, P<0.01).

**Conclusions:**

Osteoporotic postmenopausal women had a significant increase in serum lipid profile and BMI. Moreover, we found a positive link between women with cardiovascular diseases and stroke.

## Introduction

**Figure 1:**
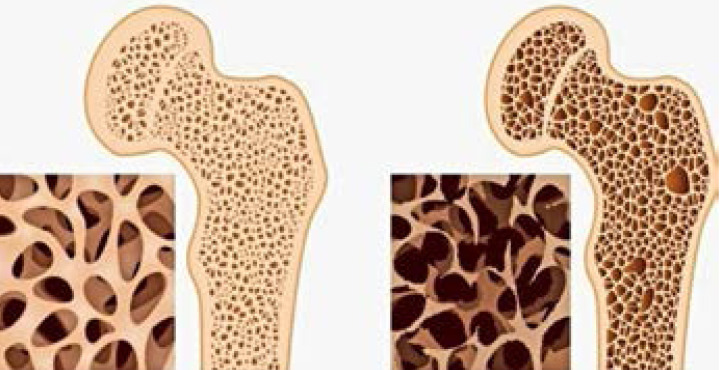
Osteoporosis inside the femoral bone.

**Figure 2:**
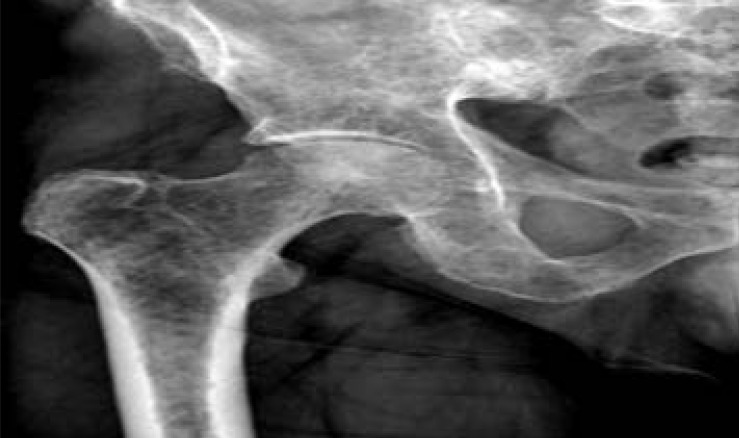
Plain X-ray image of right hip joint showed osteoporosis inside the head of femoral bone.

Epidemiological studies suggested a relation between cardiovascular diseases and osteoporosis.^1^, ^2^ The intravascular deposition of lipids is a strong risk factor for cardiovascular disease. Studies evaluating the relationship between lipid parameters and bone mineral density (BMD) in healthy adults and those with metabolic syndrome revealed inconsistent results.^3^, ^4^ Most of these studies were performed in women more than men, mainly in adolescents.^3^, ^5^, ^6^ Osteoporosis is one of the most common systemic skeletal disorders characterized by micro-architectural changes and low bone mass which increase the risk of bone fracture, (Figure 1).^7^ The fracture risk depends on bone strength, which is determined by the bone quality and Bone Mineral Density (BMD).^8^,^9^ Moreover, osteoporosis is considered as a metabolic bone disorder accompanied by low mass and weakness of the bones. Plain x-ray images can help the clinicians in the diagnosis of osteoporosis, (Figure 2). Some studies revealed a relationship between dyslipidaemia and low bone mineral density; while other studies found no relationship between total serum cholesterol levels and bone mineral density (BMD).^10^, ^5^ Osteoporosis is a major health problem in postmenopausal women and is associated with a high risk of cardiovascular disease and stroke due to raised atherogenic lipid levels.^11^ Body weight is one of the strongest positive predictors of bone mass. There is a positive link between body weight and bone mass in all age groups.^12^, ^13^ Because of the controversy in the previous studies and lack of data in Sudanese postmenopausal women, we investigated the relationship between serum lipid profile, BMI and osteoporosis in postmenopausal women.

## Materials and Methods

This was a prospective analytical case-control hospital-based study conducted in Khartoum north hospital at Khartoum city, capital of the Sudan from April 2017 to March 2018. Two hundred postmenopausal women were enrolled in this study. The age and sex matched, one hundred osteoporosis postmenopausal women as the case group and one hundred non-osteoporosis postmenopausal women as a control group.

### Inclusion and exclusion criteria

Postmenopausal women with an osteoporosis group and postmenopausal women without osteoporosis group were included in this study. Obese women, those with malignant disease, diabetes mellitus, thyroid diseases, parathyroid diseases, adrenal glands diseases, chronic renal failure, inflammatory arthritis, statins usage in the treatment of dyslipidaemia, corticosteroids, hormones and diuretics for more than three months, secondary osteoporosis due to endocrine diseases, gastrointestinal tract diseases such as (Crohn's disease, malabsorption), peptic ulcer surgery, chronic liver disease and osteoporosis induced by medications were excluded from the study.

### Data collection and sampling

The blood samples were taken from a peripheral vein after twelve hours of fasting and were immediately centrifuged at 4°C for 10 min to obtain serum. The fluorescence was measured by automated spectrofluorometer (Mandry, Germany) at 350 nm (Ex) / 420 nm (Em). The obtained values with the usage of enzymatic methods were ascribed the lipid profiles in the serum.

### Data analysis

SPSS version 16 was used for data analysis. The data are presented as the (mean ± standard deviation). The t-test was used to compare the lipid profile and BMI between the study and control group. P-value of <0.05 was considered statistically significant

## Results

The result data of BMI, serum total cholesterol, triglyceride, HDL and LDL in case group and control group were showed in (Table 1) respectively.

**Table 1:** Serum lipid profile among study population

Parameters	Case group No.=100	Control group No.=100	P-value
**BMI**	24.846±-2.1647	25.378 ±3.8115	0.226
**T-CH**	251.190±27.0135	187.990 ± 26.611	0.000
**TG**	168.790 ±45.774	139.360±20.290	0.000
**HDL**	50.620 ± 7.174	49.480 ±4.659	0.184
**LDL**	166.868 ±28.978	111.667 ±28.0045	0.00

**Figure 3:**
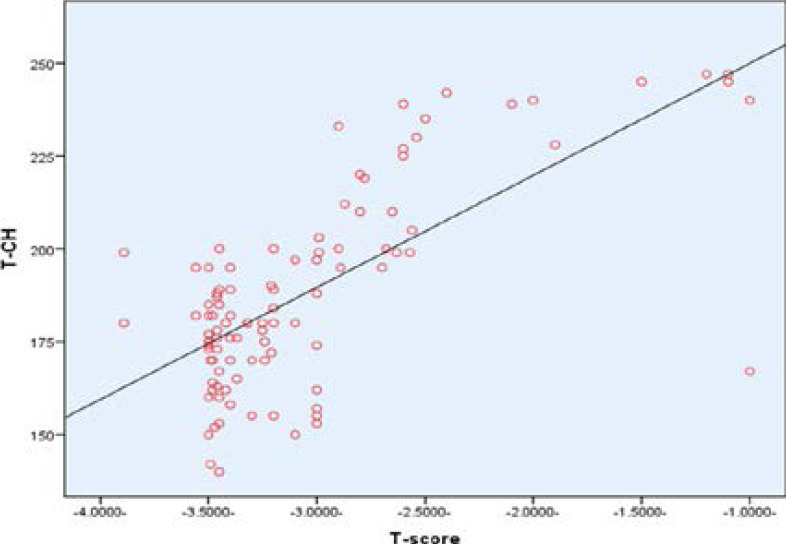
Correlations between TC and BMD (r= 0.832, P<0.01).

**Figure 4:**
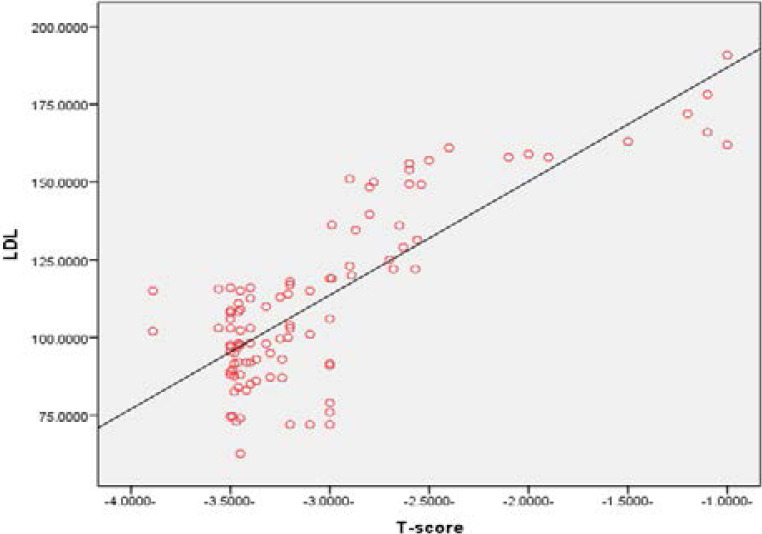
Correlation between LDL and BMD (r = 0.782, P<0.01).

**Figure 5:**
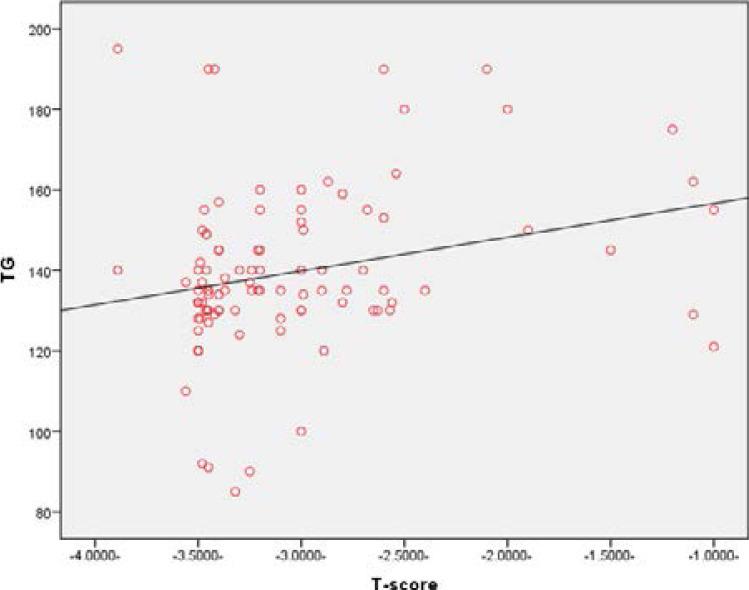
Correlation between TG and BMD (r = 0.72, P<0.01).

In addition, all serum lipid profiles significantly increased (p=0.000) in the case group compared to the control group, except serum HDL was insignificant different between the case and control group and also BMI was insignificantly difference between case and control group, (Table 1). Pearson's correlation showed a positive correlation between BMD and serum total cholesterol (r= 0.832, P<0.01), (Figure 3). Also, the Pearson's correlation revealed a positive correlation between serum LDL and BMI (r = 0.782, P<0.01), (Figure 4). In Pearson's correlation, there was a positive correlation between serum triglyceride (r = 0.72, P<0.01), (Figure 5).

## Discussion

Because of the controversy on the previous studies and lack of data in Sudanese postmenopausal women, this study investigated the relationship between serum lipid profile, BMI and osteoporosis in postmenopausal women. Two hundred postmenopausal women were enrolled in the study. One hundred osteoporosis postmenopausal women as case group and one hundred non-osteoporosis postmenopausal women as a control group. In this present study, all serum lipid profile was significantly increased in osteoporotic postmenopausal women compared to the control group except serum HDL showed the insignificant difference between case and control group. Also, BMI showed an insignificant difference between the case and control group. Pearson's correlation found a positive correlation between BMD, total serum cholesterol (TC), serum LDL and serum triglyceride (TG). Dyslipidaemia has been associated with BMD in some studies, but other studies revealed no relationship between total serum cholesterol levels and bone mineral density.^10^, ^5^ A positive association between atherosclerotic CVD and osteoporosis was supported by epidemiological studies.^14^, ^15^ Researchers also showed BMD in postmenopausal women was quantitatively associated with high lipid levels in the blood.^16^, ^5^

This study disagrees with Li et al.^17^ who reported that HDL was positively correlated with postmenopausal osteoporosis, but not LDL, TG and TC. Moreover, Sivas et al.^18^ supported this study and found a positive correlation of LDL, TC and TG with postmenopausal osteoporosis. Furthermore, our study disagrees with Wang et al.^19^ who reported that a negative correlation between LDL, TC in postmenopausal osteoporosis, but there was no significant correlation between HDL and TG in postmenopausal osteoporosis. The dyslipidaemia increases after menopause. A significant increase in TC, LDL, and TG levels has been demonstrated. HDL data have been controversial. Some authors indicated a lack of any change in HDL values, while others reported decreased or increased HDL levels.^20^–^25^ Previously, TC was thought to be associated with cardiovascular diseases and osteoporosis, and subsequent studies investigating the correlation between TC, LDL, TG, and BMD were performed. However, these studies yielded varying outcomes. Some studies found no relationship between them, while others reported a positive or negative correlation.^26^–^30^

In this study the TC, LDL, TG levels were higher in the control group compared with the osteoporotic group and there is a positive correlation with BMD. Lipid disorders have been associated with BMD in some studies.^31^ The mechanism of this relationship may be directly related to the cholesterol biosynthetic pathway which determines cholesterol levels and contributes to the activity of the osteoclast.^32^ Beneficial effects of lipid reducing drugs such as statins on BMD has been seen in most of previous studies.^33^, ^31^ Furthermore, these findings proposed the probable association between serum lipid profile and BMD especially among patients with increased risk of osteoporosis other than healthy persons.^34^, ^18^

## Conclusions

Osteoporotic postmenopausal women had a significant increase in serum lipid profile and BMI. Moreover, we found a positive link between these women with cardiovascular diseases and stroke.

## Abbreviations

BMI: body mass index
BMD: bone mass density
CVD: cardiovascular disease
HDL: high density lipoprotein
LDL: low density lipoprotein
TC: total cholesterol
TG: triglyceride
SPSS: statistical package for social sciences
